# Dominant *Candidatus* Accumulibacter phosphatis Enriched in Response to Phosphate Concentrations in EBPR Process

**DOI:** 10.1264/jsme2.ME17020

**Published:** 2017-09-27

**Authors:** Awaluddin Nurmiyanto, Hiroya Kodera, Tomonori Kindaichi, Noriatsu Ozaki, Yoshiteru Aoi, Akiyoshi Ohashi

**Affiliations:** 1 Graduate School of Engineering, Hiroshima University 1–4–1 Kagamiyama, Higashi-Hiroshima 739–8527 Japan; 2 Department of Environmental Engineering, Islamic University of Indonesia (UII) Jl. Kaliurang Km 14, Sleman, Yogyakarta 55581 Indonesia; 3 Graduated School of Advanced Sciences of Matter, Department of Molecular Biotechnology, Hiroshima University 2–313 Kagamiyama, VBL building–402, Higashi-Hiroshima, 739–8527 Japan

**Keywords:** *Candidatus* Accumulibacter phosphatis, enhanced biological phosphorus removal (EBPR), microbial diversity, phosphate concentration, polyphosphate-accumulating organisms (PAOs)

## Abstract

*Candidatus* Accumulibacter phosphatis (Accumulibacter), which plays an important role in enhanced biological phosphorus removal in wastewater treatment plants, is phylogenetically classified into two major types (Types I and II). Phosphate concentrations affect the Accumulibacter community of the biomass enriched in treatment plants. Therefore, in the present study, Accumulibacter enrichments were conducted using a down-flow hanging sponge reactor under five conditions and a wide range of controlled phosphate concentrations in order to investigate how phosphate governs the community. We found that excessive phosphate levels inhibited Accumulibacter activity, that this inhibitory effect was greater for Type II. In addition, the affinity of Type II for phosphate was higher than that of Type I. Type IIA-B dominated at a phosphate concentration less than 5 mg P L^−1^, while Type IA was dominant at 50 and 500 mg P L^−1^. These patterns of enrichment may be explained by an inhibition kinetics model.

The enhanced biological phosphorus removal (EBPR) process has been widely employed in full-scale wastewater treatment plants (WWTP) for more than three decades (6, 36, 40), and is recognized as an economic and environmentally friendly method for the removal and recovery of phosphorus from sewage (32). The performance of the EBPR process is influenced by the abundance and activity of polyphosphate-accumulating organisms (PAOs) enriched in the reactor. PAOs have the ability to take up large amounts of phosphate and store it intracellularly as polyphosphate (poly-P) (29), after which phosphorus removal may be accomplished by withdrawing excess sludge containing PAOs from the process.

None of the PAOs responsible for EBPR have been isolated and cultivated to date. Nevertheless, intensive investigations on EBPR have confirmed that the important members of PAOs are affiliated with the *Rhodocyclus* group in *Betaproteobacteria* and have been named “*Candidatus* Accumulibacter phosphatis” (hereafter referred to as Accumulibacter) (8, 13, 17). Previous studies on Accumulibacter revealed that they may be phylogenetically divided into two major types (Types I and II) based on their polyphosphate kinase (*ppk1*) genes (14, 27). Furthermore, Type I is classified into five clades (IA to IE), while Type II originally contained seven clades (IIA to IIG), but was recently expanded to include two additional clades (novel IIH and II-I) (24).

Various clades of Accumulibacter have been detected in laboratory-scale EBPR experiments and full-scale WWTPs; however, investigations on dominant Accumulibacter clades have produced different findings. Martín *et al.* (25) reported that the dominant Accumulibacter clades were IA and IIA in a laboratory-scale experiment. The same findings were obtained in other studies by He *et al.* (14) and Welles *et al.* (38, 39). In contrast, Clades IIA, IIB, and IIC were found to be dominant in a Danish WWTP (2) and WWTP in the United States (14). Clade IID was also identified as dominant in several WWTPs (24). Unfortunately, it currently remains unclear why the dominant Accumulibacter type and clade vary. Therefore, the community structures of Accumulibacter are affected by regional differences as well as variations in wastewater characteristics, system configurations, and operational conditions (15).

Welles *et al.* (39) showed that Accumulibacter Type II was dominant at a relatively low phosphate concentration, while Mao *et al.* (24) reported that Accumulibacter Type I was dominant at higher influent phosphate concentrations, and also that a correlation existed between the relative abundance of each Accumulibacter clade and influent total phosphorus, but not geographical factors. Phosphate concentrations strongly influence the Accumulibacter community structure in EBPR processes, and may be an important factor affecting the dominant Accumulibacter type and clade. However, the relationship between phosphate concentrations and the dominant Accumulibacter type is unclear despite the large amount of information available. This uncertainty may be attributed to the phosphate concentrations reported to date being based on values measured in the influent, but not in the reactors. In addition, phosphate concentrations in a reactor generally fluctuate during the aerobic period, particularly during intermittent aerobic/anaerobic sequencing batch operation (1, 25, 38).

In the present study, we tested the following hypothesis: if PAOs may be enriched while maintaining stable phosphate concentrations in the reactor throughout aerobic periods, it will be possible to clearly identify the relationship between phosphate concentrations and the dominant Accumulibacter type. In order to accomplish this, PAO enrichment experiments were conducted under controlled phosphate concentrations. In addition to testing the aforementioned hypothesis, the data generated were used to investigate the relationship between the ecology of PAOs and EBPR. A down-flow hanging sponge (DHS) reactor with high influent flow was employed to maintain a constant aerobic phosphate concentration. This system employs sponge materials with an excellent biomass retention ability (5, 23) that are also capable of cultivating slow-growing bacteria (7, 19) and PAOs (20). Although the biomass on the sponge material is a type of biofilm, the difference in substrate concentrations between outside and inside the biofilm is negligible because the substrate is transported deep inside not only by diffusion, but mostly from the water current under down-flowing conditions. The employment of a conventional EBPR system is impossible for achieving the objective of the present study because of the difficulties associated with maintaining a constant phosphate concentration in the reactor during the aerobic period.

## Materials and Methods

### Reactor and operational conditions

**Fig. 1:**
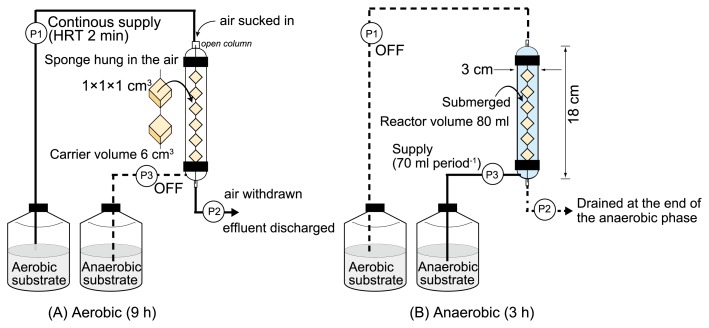
DHS reactor schematic with substrate flow under alternate aerobic (A) and anaerobic (B) conditions.

Five identical DHS reactors were used for the enrichment of PAOs at five different phosphate concentrations. Each reactor consisted of an 80-mL cylindrical glass column containing a string of six polyurethane sponge cubes (each 1×1×1 cm^3^, total volume 6 cm^3^) connected to each other diagonally in a series as biofilm carriers (Fig. 1). In order to establish PAO-enriched biofilms on the sponge cubes, biofilm carriers were exposed to alternating aerobic and anaerobic conditions. All of the reactors were operated at a cycle time of 12 h (9 h aerobic and 3 h anaerobic). The aforementioned operation according to Kodera *et al.* (20), with slight modifications, was conducted on the five reactors (Runs 1 to 5) at different aerobic influent phosphate concentrations. All experiments were conducted in a room with the temperature controlled at 20°C. Prior to the experiment, the sponge carriers were soaked in diluted activated sludge (PAO population fraction <1% of bacteria) obtained from the Higashi-Hiroshima sewage treatment plant in Japan in order to facilitate inoculation.

During the aerobic phase, phosphate-containing water (aerobic substrate) was supplied to the top of the reactor at a flow rate of 3.24 L d^−1^, corresponding to a 2-min hydraulic retention time (HRT) based on the volume of the sponge. Air was provided from the top of the reactor by sucking using a pump (P2), which also worked to drain the effluent and withdraw the sucked air simultaneously at a flow rate of 32.4 L d^−1^. The aerobic substrate containing minerals was applied at phosphate concentrations of 0.05, 0.5, 5, 50, and 500 mg L^−1^ for Runs 1 to 5, respectively, with a 0.173/0.827 molar ratio of K_2_HPO_4_ and KH_2_PO_4_ to achieve pH 7.5 (Table 1). The composition of minerals (mg L^−1^) was NH_4_Cl (3.38), CaCl_2_·2H_2_O (14), MgSO_4_·7H_2_O (90), ethylenediaminetetraacetic acid 2Na (3), FeCl_3_·6H_2_O (0.45), H_3_BO_3_ (0.045), CuSO_4_·5H_2_O (0.009), KI (0.054), MnCl_2_·4H_2_O (0.036), Na_2_MoO4·2H_2_O (0.018), ZnSO_4_·7H_2_O (0.036), NiCl_2_·6H_2_O (0.024), and CoCl_2_·6H_2_O (0.045).

During the anaerobic phase, the reactor was filled with 70 mL of an anaerobic substrate containing organic materials for 3 h, after which the solution was drained. The anaerobic substrate of 200 mg COD L^−1^ contained 146 mg L^−1^ sodium acetate (100 mg COD L^−1^), 92 mg L^−1^ sodium propionate (100 mg COD L^−1^), and the same minerals.

### Water quality analysis

Effluents from the aerobic and anaerobic periods were routinely collected once a week in order to monitor phosphate uptake-release and COD consumption. Prior to analyses, the collected samples were filtered using membranes with a pore size of 0.45 μm (Advantec, Tokyo, Japan). Phosphate concentrations and COD were measured using a HACH water quality analyzer (DR-2800, HACH, Loveland, CO, USA) according to the method numbers 8007 and 10173 in the manufacturer’s instructions, respectively.

### Fluorescent *in situ* hybridization (FISH)

Biomass sampling was conducted at the end of the anaerobic phase on day 85 when phosphate removal activity appeared to nearly reach a plateau for all runs. Each biomass was collected by squeezing the sponge carriers several times in phosphate buffer saline (PBS) solution. In the FISH analysis of the five biomass samples, fixation and hybridization were performed as previously described (20) using 16S rRNA-targeted oligonucleotide probes with 4′,6-diamidino-2-phenylindole (DAPI) staining for polyphosphate. Specifically, EUBmix (EUB338, EUB338-II, and EUB338-III) was applied to evaluate all bacteria (4, 10), PAOmix (PAO462, PAO651, and PAO846) was used for Accumulibacter (8), GAOmix (GAOQ431 and GAOQ989) for *Gammaproteobacteria*-related glycogen-accumulating organisms (GAOs) (9), and DF1mix (TFO_DF218, and TFO_DF618) (40) and DF2mix (DF988 and DF1020) (28) for *Defluviicoccus vanus* clusters I and II-related GAOs, respectively. The population fractions of the microbes detected by FISH were assessed using the method described by Kodera *et al.* (20).

### Phylogenetic analysis of Accumulibacter

DNA was extracted from the five biomass samples using a Fast DNA spin kit (MP Biomedicals, Irvine, CA, USA) according to the manufacturer’s instructions. Polymerase chain reaction (PCR) amplification of the Accumulibacter *ppk1* fragment was performed using the ONE Shot LA PCR MIX (Takara Bio, Otsu, Japan) with the following primers: ACCppk1_254f (5′-TCACCACCGACGGC AAGAC-3′) and ACCppk1-1376r (5′-ACGATCATCAGCATCTT GGC-3′) (26). The PCR conditions used were as follows: initial denaturation at 94°C for 5 min, followed by 25–30 cycles at 94°C for 30 s, at 68°C for 30 s, and at 94°C for 72 s, and then a final extension at 72°C for 4 min.

The amplified *ppk1* fragments were subsequently purified using a QIAquick PCR Purification Kit (Qiagen, Hilden, Germany), then cloned using a TOPO XL PCR Cloning kit (Invitrogen, Carlsbad, CA, USA) according to the manufacturers’ instructions. Between 13 and 24 clones were randomly selected from each clone library and subjected to sequencing at the Dragon Genomics Center (Takara Bio, Yokkaichi, Japan). The sequences obtained were then grouped into operational taxonomic units (OTUs) based on the 99% identity of DNA sequences within each library. OTUs were subsequently aligned with *ppk1* genes available in GenBank using BLAST (http://blast.ncbi.nlm.nih.gov) (3) to construct a phylogenic tree by employing the neighbor joining method within MEGA 5 (37). OTUs were classified based on previously assigned Accumulibacter clades (14). The *ppk1* gene sequence data of 14 OTUs were deposited in the GenBank/EMBL/DDBJ databases under accession numbers LC214547 to LC214560.

## Results

### Enrichment of PAOs in the DHS reactor

**Fig. 2:**
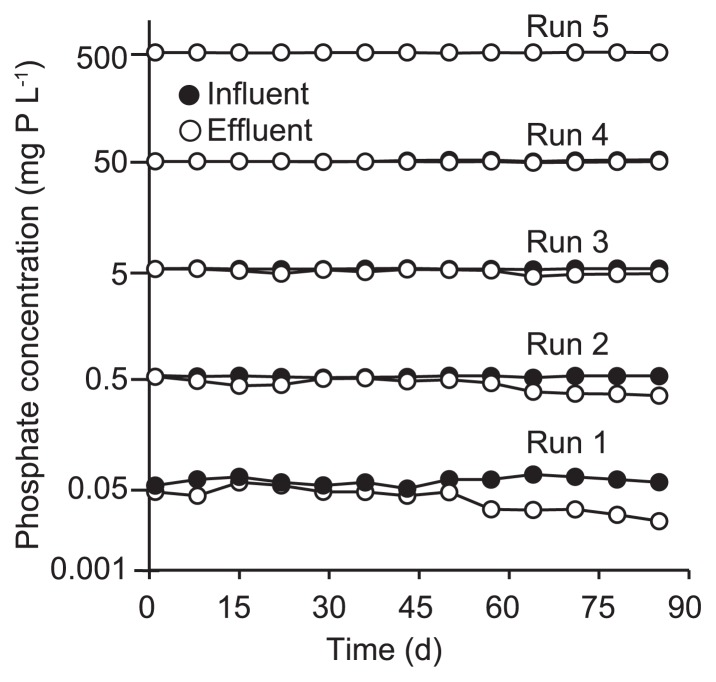
Time course of phosphate concentrations in the aerobic phase.

**Fig. 3:**
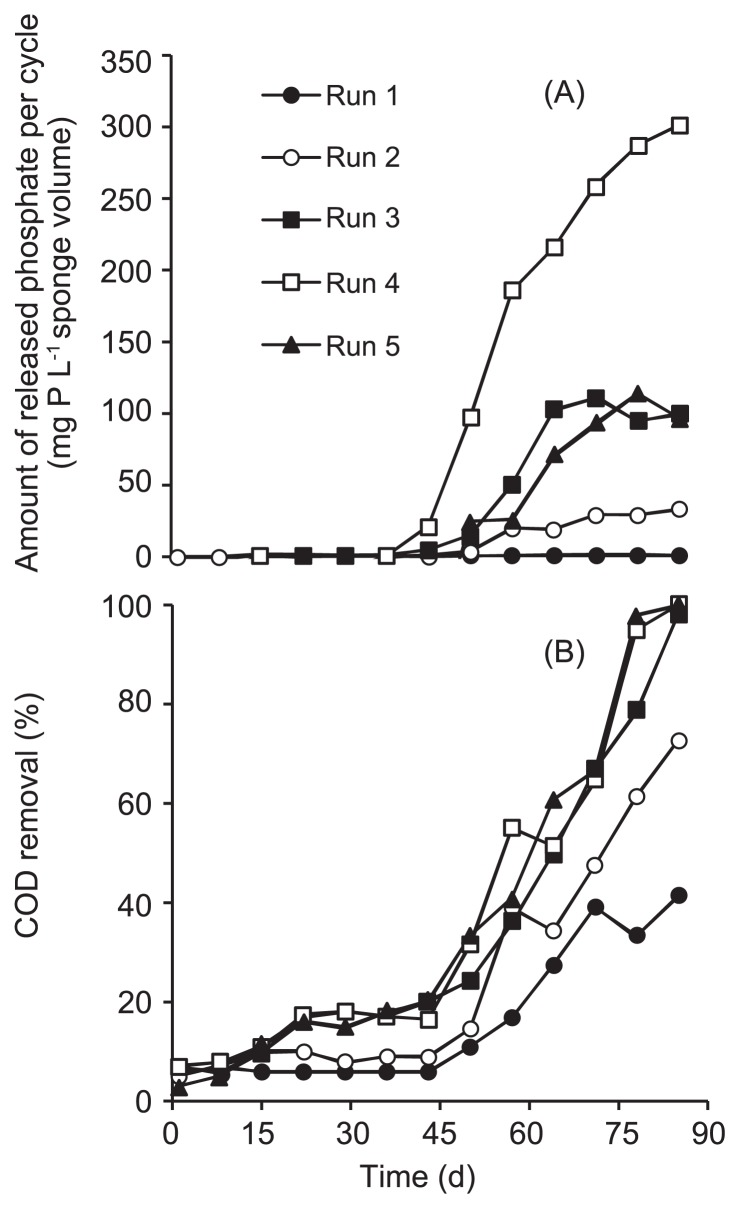
Time courses of (A) phosphate released and (B) COD consumed during the anaerobic phase.

The five DHS reactors were successfully operated for 85 d at each phosphate concentration. The removal of phosphate observed during the aerobic periods gradually increased for all Runs, except Run 5, in which the detection of phosphate removal was very difficult because of the influent phosphate concentration of 500 mg P L^−1^ (Fig. 2). During the anaerobic period, phosphate was released from the biofilm carrier into the bulk anaerobic substrate during all runs. Fig. 3A shows the time course of the amount of released phosphate calculated per cycle using Eq. S1 (see Supplementary information), in which phosphate that remained in the sponge at the beginning of anaerobic phase is taken into account, and the phosphate concentration at the end of the anaerobic phase (Fig. S1). Released phosphate increased around 40 d after the operation for all Runs, while the amounts of phosphate on day 85 appeared to increase with run number, except in Run 1. The organic substrate was consumed during the anaerobic period (Fig. 3B). On the same day, the substrate of 200 mg COD L^−1^ was almost completely consumed at the end of the anaerobic period in Runs 3–5; however, approximately 60% (120 mg COD L^−1^) and 35% (70 mg COD L^−1^) remained in Runs 1 and 2, respectively. The observed P removal during aerobic periods coupled with its release and the consumption of COD in the anaerobic period indicates that PAOs were successfully enriched for all runs at influent P concentrations of 0.05–500 mg P L^−1^.

### Phosphorus uptake and release activity of the enriched biomass

**Fig. 4:**
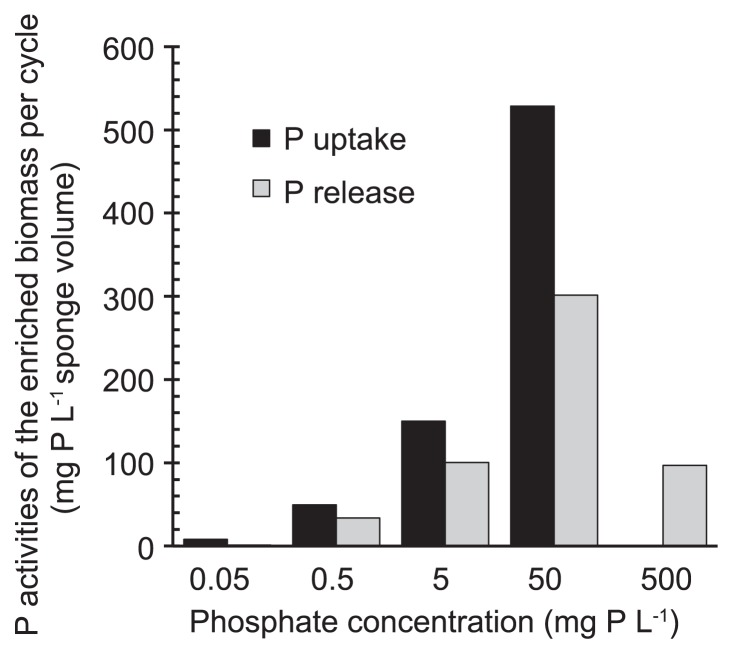
Phosphate uptake and release of the PAO-enriched biomass on day 85.

The enrichment condition strongly affected the phosphorus activities of the PAO-enriched biomass (Fig. 4). The specific rate of phosphorus uptake based on sponge volume during the aerobic period on day 85 slightly increased with an influent P concentration up to 50 mg P L^−1^. Similar results were obtained for the release of phosphorus during the anaerobic period. The specific rates of phosphorus uptake and release were expected to be similar if biomass growth reached a plateau. In Run 4, in which the influent P concentration was 50 mg P L^−1^, a significant gap was observed between the two specific rates. The cause of this gap was derived from not accounting for the amount of phosphorus in the detached and discharged biomass, which was unmeasured, in the release rate. In Run 5, P activities were reduced. The very high influent phosphate concentration of 500 mg P L^−1^ was expected to inhibit PAO activity, but not that of other bacteria because the consumption of COD did not decrease. In contrast, COD was incompletely consumed in Runs 1 and 2, indicating that the low concentration of phosphate weakened the activity of not only PAOs, but also other microbes.

### Microbial community structure by FISH

**Fig. 5:**
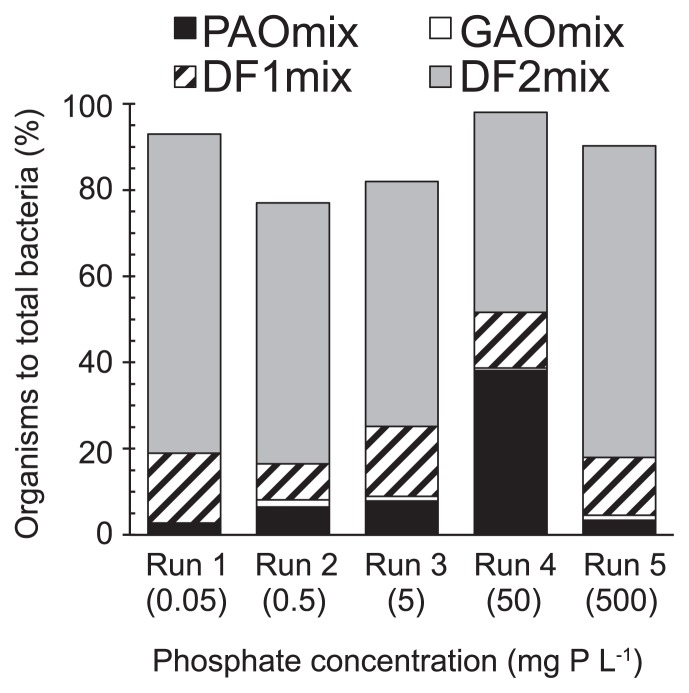
Microbial community structure of the biomass on day 85. The percentage of each microbe over total bacteria was assessed by FISH using EUBmix, DF1mix, and DF2mix probes.

The FISH analysis with DAPI staining revealed that Accumulibacter was enriched for all runs. Representative FISH images are shown in Fig. S2. The other PAOs were not detected because DAPI-stained cells completely overlapped with the fluorescence signal of the PAOmix probe. The abundance of Accumulibacter differed according to the phosphate concentration in the aerobic phase (Fig. 5). In Run 1, Accumulibacter accounted for only approximately 2% of all bacteria on day 85. The abundance of Accumulibacter increased with influent phosphate concentrations up to 50 mg P L^−1^, and were dominant in Run 4, accounting for approximately 36% of all bacteria. The levels of Accumulibacter decreased in response to the high phosphate concentration of 500 mg P L^−1^ in Run 5. These results were consistent with those of phosphorus uptake and release (Fig. 4), suggesting that the phosphorus activities of the biomass were strongly related to the abundance of Accumulibacter. In addition, these results showed that high phosphate concentrations negatively affected the activities and growth of Accumulibacter.

GAOs were more highly enriched than Accumulibacter, accounting for more than 50% of all bacteria in all runs (Fig. 5). Competition between Accumulibacter and GAOs may be affected by several conditions in the EBPR process, including pH (12, 30), temperature (22), the type of carbon source (31), and phosphate to carbon ratio in the influent (18, 21). Under these enrichment conditions, the most dominant GAO group was *Defluvicoccus* spp., which maintained their activity, even under the high phosphate concentrations in Run 5 relative to Accumulibacter. Although a large quantity of GAOs was detected, this result did not markedly affect our objective, which was to investigate the influence of aerobic phosphate concentrations on competition among Accumulibacter clades.

### Clades of enriched PAO Accumulibacter

**Fig. 6:**
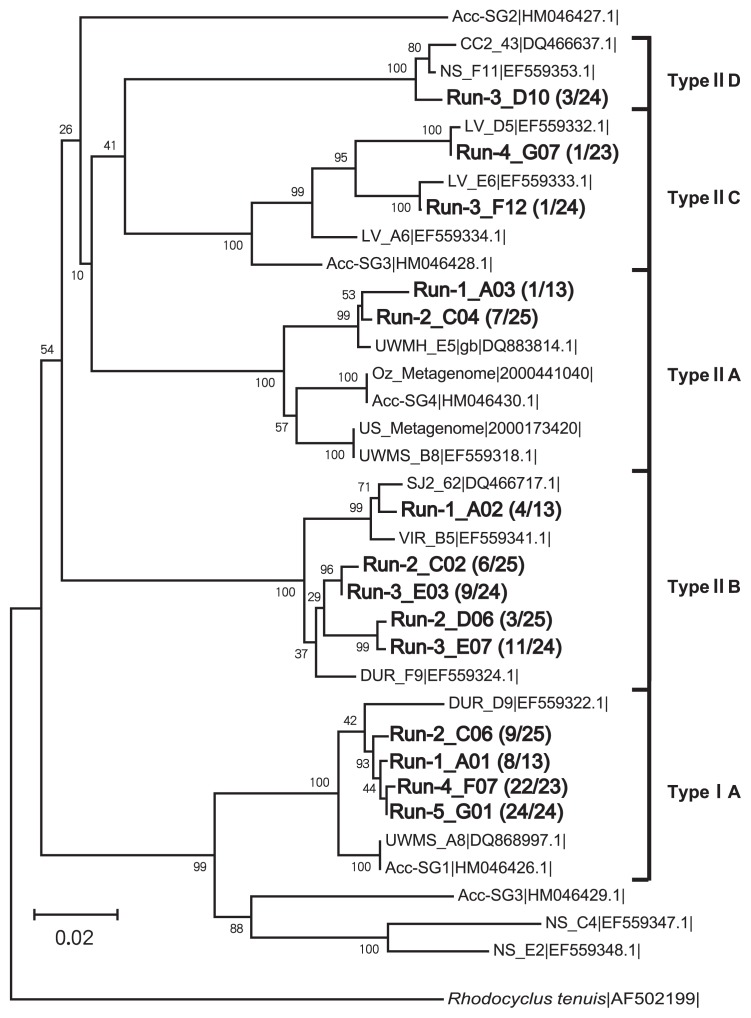
Phylogenetic tree of Accumulibacter *ppk1* gene sequences on day 85. The first number in the parentheses indicates the number of clones belonging to this OTU, and the second number is the total number of *ppk1* clones retrieved in each run.

A clone library based on the *ppk1* gene was constructed to reveal the fine-scale population structures of Accumulibacter enriched under five different phosphate concentrations. The phylogenetic tree (Fig. 6) showed that Types I and II were both retrieved on day 85. Only one clade of IA was detected in Type I. However, four clades of IIA to IID were observed in Type II, suggesting that specific Accumulibacter lineages preferable to each condition, but not all of the clades, are enriched under the defined enrichment conditions.

**Fig. 7:**
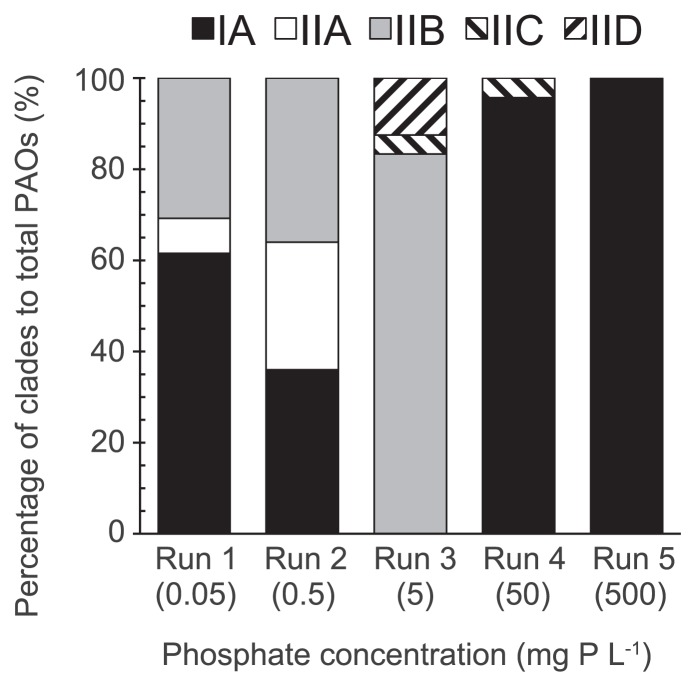
Composition of Accumulibacter in samples on day 85 based on the number of *ppk1* clones.

As shown in Fig. 7, most clones were affiliated with clade IA (62% of the total; 8 out of 13 clones) at very low phosphate concentrations (Run 1). The remaining clones belonged to clade IIA and IIB of Accumulibacter. The cultivated Accumulibacter was composed of three clades, not a single one. In Run 2, the clade composition was identical to that in Run 1, whereas the composition ratio was different. Clade IA was less than that in Run 1, whereas the ratio of clades IIA and IIB was higher. In Run 3, which had a phosphate concentration of 5 mg P L^−1^, clades IA and IIA disappeared completely, while clade IIB dominated 83% (20 out of 24 clones) of the Accumulibacter population, and clades IIC and IID appeared. Clade IA reappeared at a higher phosphate concentration of 50 mg P L^−1^ (Run 4), and became the dominant Accumulibacter. The population of Accumulibacter consisted entirely of clade IA in Run 5, in which the phosphate activity of Accumulibacter was depressed. Overall, these results indicate that phosphate significantly governed the community of Accumulibacter through a complex mechanism.

## Discussion

Our results demonstrated that Accumulibacter Type I and/or II may be enriched in a wide range of aerobic phosphate concentrations of 0.05 to 500 mg P L^−1^, and that phosphate concentrations appear to strongly affect the dominant Accumulibacter Type in the biomass. Even at an extremely low phosphate concentration of 0.05 mg P L^−1^, Accumulibacter was enriched, although the population size was markedly smaller than that of the other microbes, whereas Type II was dominant at a phosphate concentration of 0.05 mg P L^−1^. The results of the Accumulibacter community analysis were used as a benchmark because the microbial communities enriched under very low phosphate concentrations have not yet been analyzed.

At relatively low phosphate concentrations of 0.5 and 5 mg P L^−1^, Accumulibacter Type II were prevalent in our experiments. As shown in Table 2, Type II, but not Type I, was found to be dominant in most full-scale EBPRs treating domestic sewage, in which the phosphate concentration is typically less than 5 mg P L^−1^ in the reaction tanks (24). Welles *et al.* (39) also reported that Type II was dominant in a SBR with an influent of 2.2 mg P L^−1^. These previous findings on Type II dominance are consistent with the present results. Previous studies suggested that a low phosphate concentration is favorable for Type II rather than Type I (1, 38). At high phosphate concentrations of 50 and 500 mg P L^−1^, Type I dominated the communities. Welles *et al.* (38) obtained similar findings at a high influent phosphate concentration of 25 mg P L^−1^, in which Type I accounted for 98% of Accumulibacter. These results show that Type I has a faster growth rate than Type II at high phosphate concentrations. However, Type I did not always prefer higher phosphate concentrations, as indicated by the phosphorus uptake activity and Accumulibacter population size decreasing in Run 5 (Figs. 4 and 5).

At influent phosphate concentrations of 5–25 mg P L^−1^, the predominant Accumulibacter type differed (Table 2). Type I accounted for 52% and 61% of Accumulibacter in the full-scale WWTPs of Singapore (SG-SG-UP) and Leicester (UK-WL-OW), respectively. In contrast, 85% of the *ppk1* genes retrieved from a Beijing WWTP (CN-BJ-BX) were affiliated with Type II. This difference in the dominant Accumulibacter type may be explained by the phosphate concentrations in the aerobic tank because phosphate concentrations in sequencing batch reactors (SBR) will change significantly during the aerobic phase. However, actual phosphate concentrations in the aerobic tanks were not reported (24). Welles *et al.* (38) showed that the initial phosphate concentration of 40 mg P L^−1^ in the aerobic phase was reduced to zero in a very short time (30 min) during SBR operation, and there was subsequently no phosphate for a long aerobic period; 99% of Accumulibacter was Type II. In contrast, Type I was dominant after a high phosphate concentration of 140 mg P L^−1^ was reduced to 0 mg P L^−1^ over 3 h in the aerobic phase. The present study was designed to maintain stable phosphate concentrations in the reactor throughout the aerobic period. As a result, all runs were successfully performed with negligible phosphate concentration changes in the aerobic period, which means our results for the relationship between phosphate concentrations and the enriched Accumulibacter type appear to be reliable. Therefore, previous findings and the present results collectively suggest a shift in the dominant Accumulibacter from Type II to Type I at a transition phosphate concentration between 5 and 50 mg P L^−1^.

When a species of bacteria becomes dominant under cultivation conditions, it has a higher growth rate than the other bacteria in the environment. The microbial growth rate is generally dependent on the substrate concentration. Our results demonstrated that the dominant Accumulibacter was Type I, II, II, and I at 0.05. 0.5, 5, and 50 mg P L^−1^, respectively. Our results also suggested that high phosphate concentrations inhibited both types of Accumulibacter. These changes in Accumulibacter dominance may be explained by modified Michaelis-Menten kinetics with high substrate concentration inhibition (11), as shown in following equation:

$$\nu =\frac{V_{\text{max}}[S]}{(K_{m}+[S])\left(1+\frac{\left[S\right]}{K_{\text{i}}}\right)}$$

where, *ν*, *V*_max_, *S*, *K*_m_, and *K*_i_ are the specific substrate utilization rate (T^−1^), maximum specific substrate utilization rate (T^−1^), substrate concentration (mg L^−1^), half saturation constant (mg L^−1^), and inhibition constant (mg L^−1^), respectively.

The values of the kinetic parameters for Accumulibacter have not been assessed because no Accumulibacter has been isolated to date (32, 41). Therefore, we assumed the values shown in Table 3 to simulate the results of enriched Accumulibacter types by trial and error, not from calculations. This set of assumed values is one of the examples proposed to explain the phenomena.

**Fig. 8:**
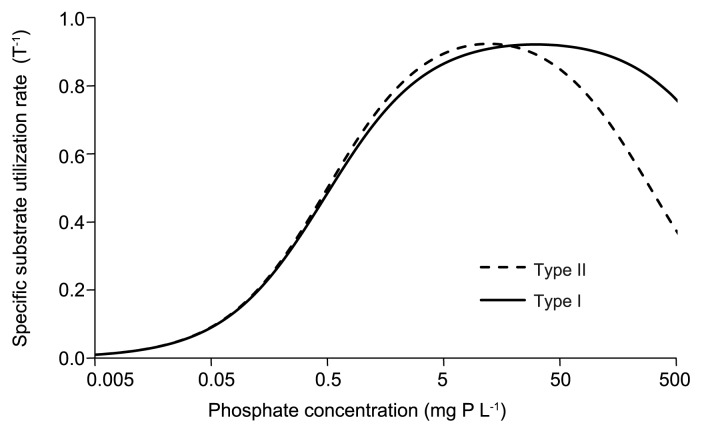
Specific substrate utilization rates of Accumulibacter Types I and II simulated using kinetic parameters listed in Table 3.

Fig. 8 shows substrate utilization rates related to phosphate concentrations drawn using the values of parameters for Types I and II. Since the substrate utilization rate of Accumulibacter is expected to correspond to their growth rates, a type of Accumulibacter having a higher substrate utilization rate than other types will be dominant. The dominant Accumulibacter types predicted from Fig. 8 matched well with the results of the present study, indicating that Types I and II had similar affinities for phosphate concentration *K*_m_ because they were simultaneously enriched at very low phosphate levels. As shown in Table 3, Type II possessed a slightly higher *V*_max_ than Type I. *K*_i_ values indicated that Type II was more strongly inhibited by high phosphate concentrations than Type I (Fig. 8).

Fourteen *ppk1* clades of IA to IE in Accumulibacter Type I and IIA to II-I in Type II have been identified to date (24). These clades have been reported to associate with the environmental habitat (16). In this study, only clades IA and IIA to IID were retrieved, even though Accumulibacter was enriched under a wide range of phosphate concentrations. Clades IB, ID, and IE have been specifically detected in saline environments such as estuary sediment (35), suggesting that Accumulibacter Type I (except clade IA) is halophilic. This may explain why these clades were not retrieved in our experiments without saline. All Type II clades have been detected from 18 WWTPs treating sewage with 2 to 10 mg P L^−1^ (24). The results of the present study combined with previous findings collectively suggest that fresh water is favorable for Accumulibacter Type II. The members of different Accumulibacter clades may have varying optimum environments. For example, Clade IIF may be mesophilic and prefer higher temperatures because its members are commonly found at 28°C to 32°C (33, 34). However, the optimum conditions of Clade IIE, IIG, IIH, and II-I, which were not detected in the present study, are not clear. Difficulties are associated with predicting which Accumulibacter clade becomes dominant based only on phosphate concentrations; therefore, further studies are needed in order to understand the ecology of Accumulibacter.

## Conclusion

The results of the present study revealed that phosphate concentrations are a significant factor influencing the dominant Accumulibacter type and population size in an enriched biomass, resulting in the phosphate removal activity of a bioreactor increasing with elevations in phosphate concentrations. However, we found that excessive phosphate concentrations inhibited Accumulibacter activity. Overall, the results of the present study suggest that changes in the dominant Accumulibacter type along with phosphate concentrations are explainable by the modified Michaelis-Menten kinetics model with strong phosphate inhibition.

## Supplementary Material



## Figures and Tables

**Table 1:** Substrate concentrations for PAO enrichment

Phase	Substrate (mg L^−1^)	Run 1	Run 2	Run 3	Run 4	Run 5
Aerobic	Phosphate	0.05	0.5	5	50	500
	COD			0		

Anaerobic	Phosphate			0		
	COD			200		

**Table 2:** Summary of Accumulibacter-type fractions and phosphate concentrations reported previously and in the present study

Accumulibacter		Operational variables									
	
Type I (%)	Type II (%)	Phosphate (mg P L^−1^)			Influent COD (mg COD L^−1^)	Temperature (°C)	pH	Carbon source	Code	Reactor type	Reference

		Influent	Aerobic started	Finished Aerobic							
61	37	0.05	0.05	0.03	200	20	7.5	50% Ace:50% Pro	RUN 1	A/O DHS (Lab)	This study
38	62	0.5	0.5	0.42	200	20	7.5	50% Ace:50% Pro	RUN 2	A/O DHS (Lab)	

1^a^	99^a^	2.2	3.1	0^b^	400	20	7	Acetate	SBR	A/O SBR (Lab)	(39)

2.4	97.6	3.9	NA	NA	102	19	NA	Domestic sewage	JP-A2O-TK	A/A/O WWTP	(24)
NA	100	3.9	NA	NA	102	19	NA	Domestic sewage	JP-STD-TK	CAS WWTP	
2.6	97.4	4	NA	NA	402	18	NA	Domestic sewage	US-GR-PC	OD WWTP	
ND	100	4.9	NA	NA	277	17	NA	Domestic sewage	CN-WH-LW	A/A/O WWTP	

17	83	5	5	4.68	200	20	7.5	50% Ace:50% Pro	RUN 3	A/O DHS (Lab)	This study

61.3	38.7	8	NA	NA	657	NA	NA	Domestic sewage	UK-WL-OW	A/A/O WWTP	(24)
52.4	47.6	9.3	NA	NA	265	27	NA	95% domestic sewage	SG-SG-UP	CAS+MBR WWTP	
14.1	85.9	9.3	NA	NA	462	16	NA	95% domestic sewage	CN-BJ-BX	A/A/O+MBR WWTP	

1^a^	99^a^	15	40	0^c^	400	20	7	Acetate	SBR-L	A/O SBR (Lab)	(38)
98^a^	ND^a^	25	140	0^d^	400	20	7.6	75% Hac:25% HPr	SBR-S	A/O SBR (Lab)	

95	5	50	50	48.8	200	20	7.5	50% Ace:50% Pro	RUN 4	A/O DHS (Lab)	This study
100	NA	500	500	500	200	20	7.5	50% Ace:50% Pro	RUN 5	A/O DHS (Lab)	

The percentage of the Accumulibacter fraction over the total Accumulibacter population was enumerated by the number of Accumulibacter *ppk1* gene clones and ^a^FISH.

^b^; ^c^; and ^d^ are 90 min; 30 min; and 3 h, respectively, when phosphate concentrations become zero in the aerobic phase.

Abbreviations: NA (Not available); ND (Not detected); Ace (Acetate); Pro (Propionate); CA (Cassamino Acids); A/O (Anaerobic, Oxic); A/A/O (Aeorbic, Anoxic, Oxic); CAS (Conventional activated sludge); OD (Oxidation ditch); SBR (Sequence batch reactor); MBR (Membrane bioreactor), WWTP (Wastewater treatment plant).

**Table 3:** Assumed values of kinetic parameters explaining the dominance of Accumulibacter

Accumulibacter	*V*_max_ (T^−1^)	*K*_m_ (mg L^−1^)	*K*_i_ (mg L^−1^)
Type I	0.95	0.48	2,000
Type II	1.0	0.5	300
