# Supplementary material for: Dominant Candidatus Accumulibacter phosphatis Enriched in Response to Phosphate Concentrations in EBPR Process
Source: Microbes Environ. 2017 Sep 27;32(3):260–7. doi: 10.1264/jsme2.ME17020 (PMC5606696; doi:10.1264/jsme2.ME17020)
Supplement: Supplementary file 1 [file 32_260_s1.pdf]

Supplemental material for:

Dominant *Candidatus* Accumulibacter phosphatis enriched in response to phosphate concentration in EBPR process

Awaluddin Nurmiyanto[1,2], Hiroya Kodera[1], Tomonori Kindaichi[1], Noriatsu Ozaki[1], Yoshiteru Aoi[3], Akiyoshi Ohashi[1*].

[1]*Graduate School of Engineering, Hiroshima University, 1-4-1 Kagamiyama, Higashi-Hiroshima, 739-8527, Japan*

[2] *Department of Environmental Engineering, Islamic University of Indonesia (UII), Jl. Kaliurang Km 14, Sleman, Yogyakarta 55581, Indonesia*

[3] *Graduated School of Advanced Sciences of Matter, Department of Molecular Biotechnology, Hiroshima University, 2-313 Kagamiyama, VBL building-402, Higashi-Hiroshima, 739-8527, Japan*

*Corresponding author: **Akiyoshi Ohashi**

Tel.: +81 082 4247823; Fax: +81 082 4247823.

E-mail: ecoakiyo@hiroshima-u.ac.jp

List of Supplemental materials

SI. 1 Calculation of the activity of phosphate release in anaerobic phase, corresponding to the amount of released phosphate per cycle

Fig. S1 Time course of phosphate concentration at the end of anaerobic phase

Fig. S2 Representative FISH images of each biomass on day 85

Supplemental Information 1:

Calculation of the activity of phosphate release in anaerobic phase, corresponding to the amount of released phosphate per cycle.

The activity can be calculated using the data of the phosphate concentration at the end of anaerobic phase and the phosphate concentration of aerobic substrate. The influent phosphate of aerobic substrate should be taken into account because phosphate is remained in the sponge at almost the same concentration of aerobic substrate at the beginning of anaerobic phase. The calculating formula is as follows:

$$R_r = \frac{C_e \times (V_a + V_s) - (C_a \times V_a) + (C_s \times V_s)}{V_s} \quad \text{Eq. S1}$$

where, R_r: the activity of phosphate release in anaerobic phase, C_a: the phosphate concentration of anaerobic substrate (0 mg P L^{-1}), C_s: the phosphate concentration of the influent in aerobic phase, C_e: the phosphate concentration at the end of anaerobic phase, V_a: the volume of anaerobic substrate supplied at one cycle (70 ml), and V_s: the total sponge volume (6 ml).

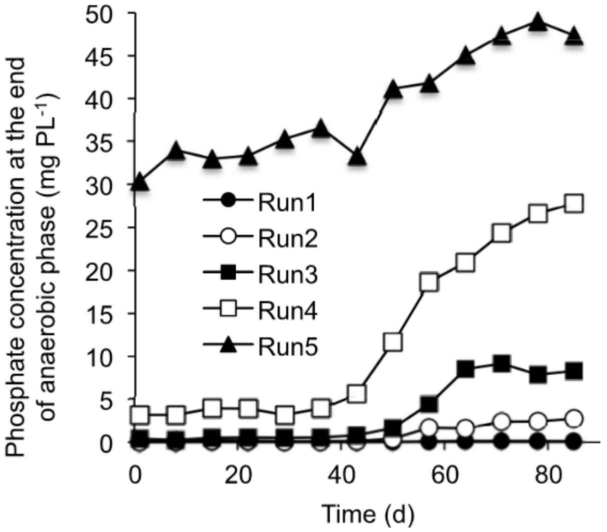

Fig. S1 Time course of phosphate concentration at the end of anaerobic phase

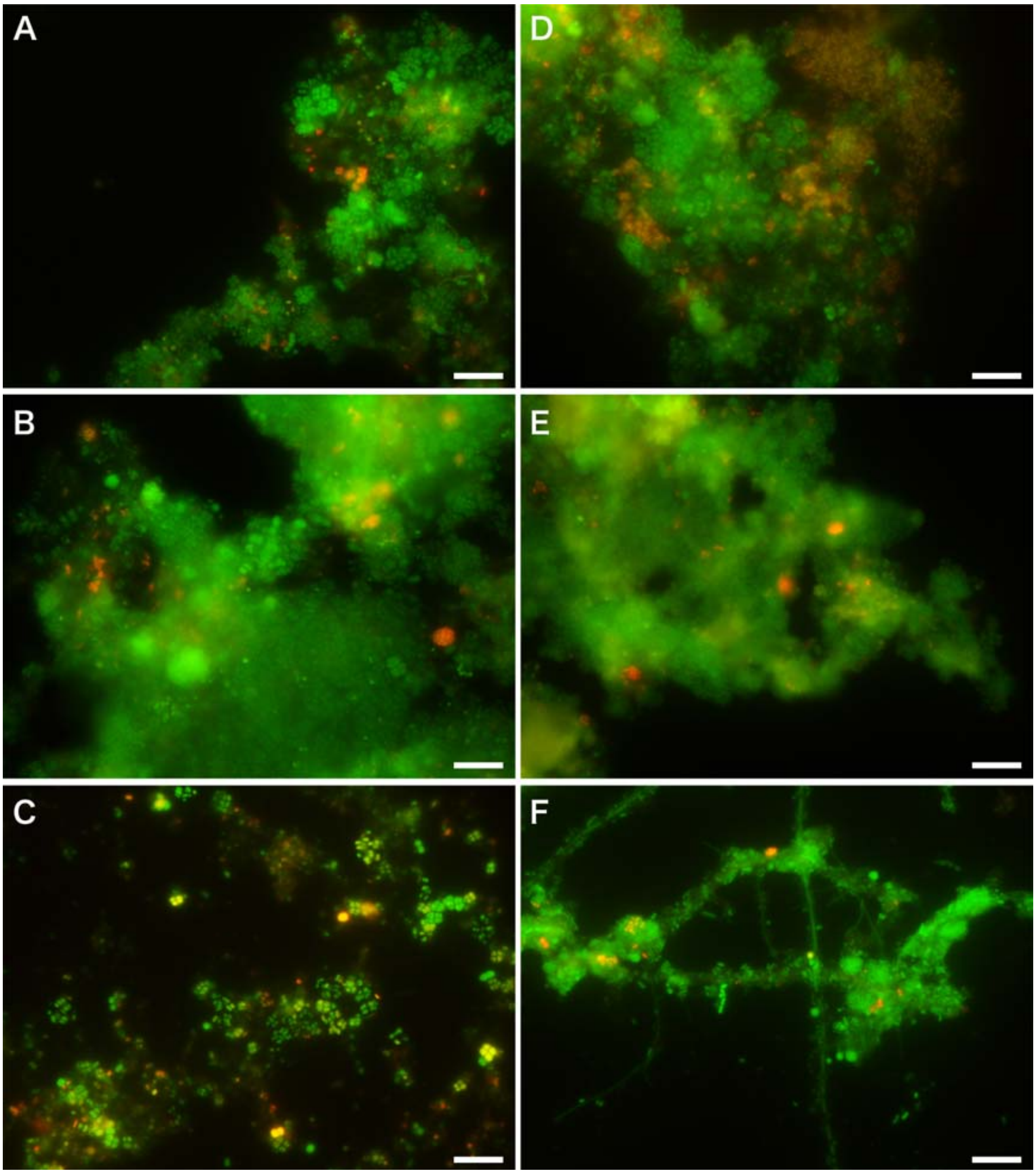

Fig. S2 Representative FISH images of each biomass on day 85. FISH images of biomass collected from Run 1 (A), Run 2 (B), Run 3 (C), Run 4 (D), Run 5 (E), and activated sludge used as the inoculum (F). FISH was performed with Alexa Fluor 488-labeled EUBmix probe (green) and Cy3-labeled PAOmix probe (red). Scale bars represent 10 μm.
